# Internet gaming disorder, attention deficit hyperactivity disorder and learning in adults: a systematic review

**DOI:** 10.3389/fpsyt.2025.1735922

**Published:** 2026-01-22

**Authors:** Xiaodeng Zhou, Juntao Chen, Jiahua Yao, Derek Tinyol Chui, Su-Kit Tang

**Affiliations:** 1 Faculty of Applied Sciences, Macao Polytechnic University, Macao, Macao SAR, China; 2 Faculty of Cultural Industry, Concord University College Fujian Normal University, Fuzhou, China; 3 Enviroforce Monitoring, Inspection and Testing Co. Ltd., Macao, Macao SAR, China

**Keywords:** adult learning group, attention deficit hyperactivity disorder (ADHD), comorbidity, internet gaming disorder (IGD)/gaming disorder(GD), systematic review

## Abstract

The comorbidity between attention deficit hyperactivity disorder (ADHD) and Internet gaming Disorder (IGD) has attracted much attention. The aim of this systematic review was to explore the specific association between ADHD and IGD/GD Adult learning group. Through the literature search of MEDLINE, PUBMED and CINAHL, a total of 14 relevant studies were included. The study found that ADHD symptoms (especially inattention and hyperactive impulsivity) were significantly associated with the incidence and severity of IGD/GD, suggesting that ADHD may be an important risk factor for IGD/GD. Individuals with comorbidity not only have higher mental health problems such as depression and anxiety, but also show lower self-control and higher emotionality. In addition, five factors have been mentioned as potentially mediating the association between adult ADHD symptoms and IGD/GD symptoms: mental health, gender difference, cultural context, gaming motivation and game type, and living habit. Furthermore, IGD/GD has a negative impact on academic performance in adults with ADHD. The results of this review contribute to a better understanding of the complex relationship between ADHD and IGD/GD adult learning group and provide new perspectives for clinical intervention and treatment.

## 1 Introduction

With the rapid development of digital technology and the widespread popularity of video games, Internet Gaming Disorder (IGD)/Gaming Disorder (GD) have gradually become global public health issues. IGD was listed as a condition for further study in the 5th edition of the Diagnostic and Statistical Manual of Mental Disorders (DSM-5) (1). Non-internet and offline games were also included (2). In 2018, IGD/GD was officially included as a mental health disorder in the 11th version of the International Classification of Diseases (ICD-11) (3). IGD/GD is closely associated with various mental health problems, including depression, anxiety, social phobia, and impulse control disorders (4, 5), leading to significant impairment or distress in personal, family, social, educational, occupational, or other important areas of functioning. For students, no matter what stage of learning, IGD/GD will cause a series of impacts on learning conditions (6, 7). Even in adulthood, IGD still has a negative effect on college students’ academic performance, especially grade point average (GPA) (8, 9).

Inattention, impulsivity, and hyperactivity are typical symptoms of individuals with attention deficit hyperactivity disorder (ADHD) (10–12). However, most of the previous studies focused on children and adolescents with ADHD, and there were few studies on adults with ADHD. A survey conducted in 2022 indicated that the prevalence of ADHD among children and adolescents worldwide is approximately 5.3%, while the prevalence among adults is less than half of this rate, at about 2.5% (13). The prevalence among adults may be underestimated due to the subjective nature of the symptoms, which share common features with various mental, behavioral, and physical disorders, making it prone to misdiagnosis as other psychiatric disorders (14), such as anxiety disorders, bipolar disorder, and borderline personality disorder (15). In the United States, the overall prevalence in the population increased from 6.1% in 1997 to 10.2% in 2016 (16), yet only 10.9% of adults with ADHD are receiving treatment (17). Adults with ADHD often exhibit a range of functional impairments, such as sluggish cognitive tempo (SCT), poor time management, executive function deficits, emotional instability, and inadequate stress coping mechanisms (18–20). These symptoms not only cause distress to the individuals themselves but also have negative impacts on their families, social circles, and work environments.

A growing body of research indicates the significant association between adult ADHD and IGD/GD. The existing literature proposes several potential shared mechanisms (21). For example, impulsivity, heightened reward sensitivity and delay aversion, and deficits in executive function or self-regulation may make immediate and frequent in-game rewards more salient to adults with ADHD, thereby increasing vulnerability to IGD/GD (4, 20, 22). In addition, comorbid symptom domains such as sluggish cognitive tempo or cognitive disengagement and emotional dysregulation may further weaken self-control in gaming contexts among adults with ADHD (23, 24). Meanwhile, co−occurring ADHD and IGD/GD can jointly impair individuals’ academic performance (25, 26).

In this context, we focus on this research gap, there remain many unresolved questions regarding the specific relationship and mechanisms between adult ADHD and IGD/GD. The purpose of this study is to explore the specific association between adult ADHD and IGD/GD comorbidity in order to help improve the well-being of individuals with comorbid adult ADHD and IGD/GD.

## 2 Method

This review was conducted in accordance with the PRISMA 2020 guideline.

### 2.1 Research question

Through this systematic review, we aim to address the following research questions (RQs):

RQ 1. Is there a direct association between Adult ADHD symptoms and the severity of IGD/GD?
RQ 2. What factors potentially mediate the relationship between adult ADHD symptoms and IGD/GD symptoms?
RQ 3. How is academic performance in adult ADHD affected by IGD/GD?

### 2.2 Data searching strategy

We searched three databases: MEDLINE, PUBMED, and CINAHL, using the keywords “ADHD” or “attention deficit hyperactivity disorder”, “Adult” or “Adults”, “Gaming Disorder” or “Video-game addiction” or “Gaming addiction” or “Internet gaming disorder”. The searching work was completed in October 2024.

### 2.3 Inclusion criteria

The inclusion criteria for the literature review included: written in English, the subjects were adults over the age of 18 (two papers included subjects aged 17, and were also considered, 27, 28), and conducted empirical studies. Exclusion criteria included: published before 2019, review articles, and low relevance (29, 30). (**Table 1**).

**Table 1:** Inclusion and exclusion criteria of data searching.

Inclusion criteria	Exclusion criteria
Language in English	Published before 2019
The subjects are adults	Review article
Empirical study	Low correlation

### 2.4 Data extraction

We extracted study characteristics (author, year, country/region, sample size and characteristics), measurement instruments (e.g., ASRS, IGDS9−SF), exposure/outcome definitions, potential mediators/moderators, and main findings. Data were extracted independently by two reviewers and cross−checked; disagreements were resolved by consensus.

### 2.5 Quality assessment (Newcastle–Ottawa Scale, NOS)

We evaluated the methodological quality of the 14 included studies using a cross−sectional adaptation of the Newcastle–Ottawa Scale (NOS). The adapted NOS comprised three domains aligned one−to−one with the table columns: Selection (Representativeness, Sample Size Justification, Non-Respondents, Exposure Ascertainment, Exposure Quality), Comparability (Comparability Core, Comparability Additional), and Outcome (Outcome Assessment, Outcome Definition, Statistics Appropriate). Each satisfied item scored 1 point (0 if not satisfied), for a maximum of 10 points (Selection 0–5; Comparability 0–2; Outcome 0–3). Two reviewers rated independently; disagreements were resolved by discussion (a third reviewer was pre−specified if needed). NOS scores were not used as exclusion criteria; rather, they informed (i) the risk−of−bias profile of individual studies; (ii) narrative emphasis and sensitivity considerations (e.g., whether clinical interview/diagnosis was used; whether age/sex/depression/gaming time were adjusted in the primary model); and (iii) qualitative, domain−level interpretation of heterogeneity. Domain scores, total scores, and the Scoring Criteria are provided in **Supplementary Table S1**.

Overall, total scores clustered at 6/10 (twelve studies scored 6, one scored 5, and one scored 7). The main weaknesses lay in Selection: most studies used convenience samples and lacked Sample Size Justification and Non-Respondents information; only three studies satisfied Exposure Quality via clinical interview/diagnosis for ADHD. Most studies met Comparability Core by adjusting ≥1 core confounder (e.g., sex/age), and a substantial proportion additionally met Comparability Additional by further adjusting key confounders (e.g., depression/anxiety, gaming time, sleep). Outcome performance was generally strong: most studies used validated instruments or clinical diagnoses for Outcome Assessment, defined thresholds/DSM–ICD criteria for Outcome Definition, and applied appropriate methods under Statistics Appropriate (e.g., regression/ANCOVA/mediation or moderation/SEM with fit indices). Accordingly, our results and discussion place greater emphasis on studies with clinical diagnosis and more comprehensive confounder control; sources of bias and methodological improvements are elaborated in Section 4.2.

## 3 Results

### 3.1 Studies selection

**Figure 1:**
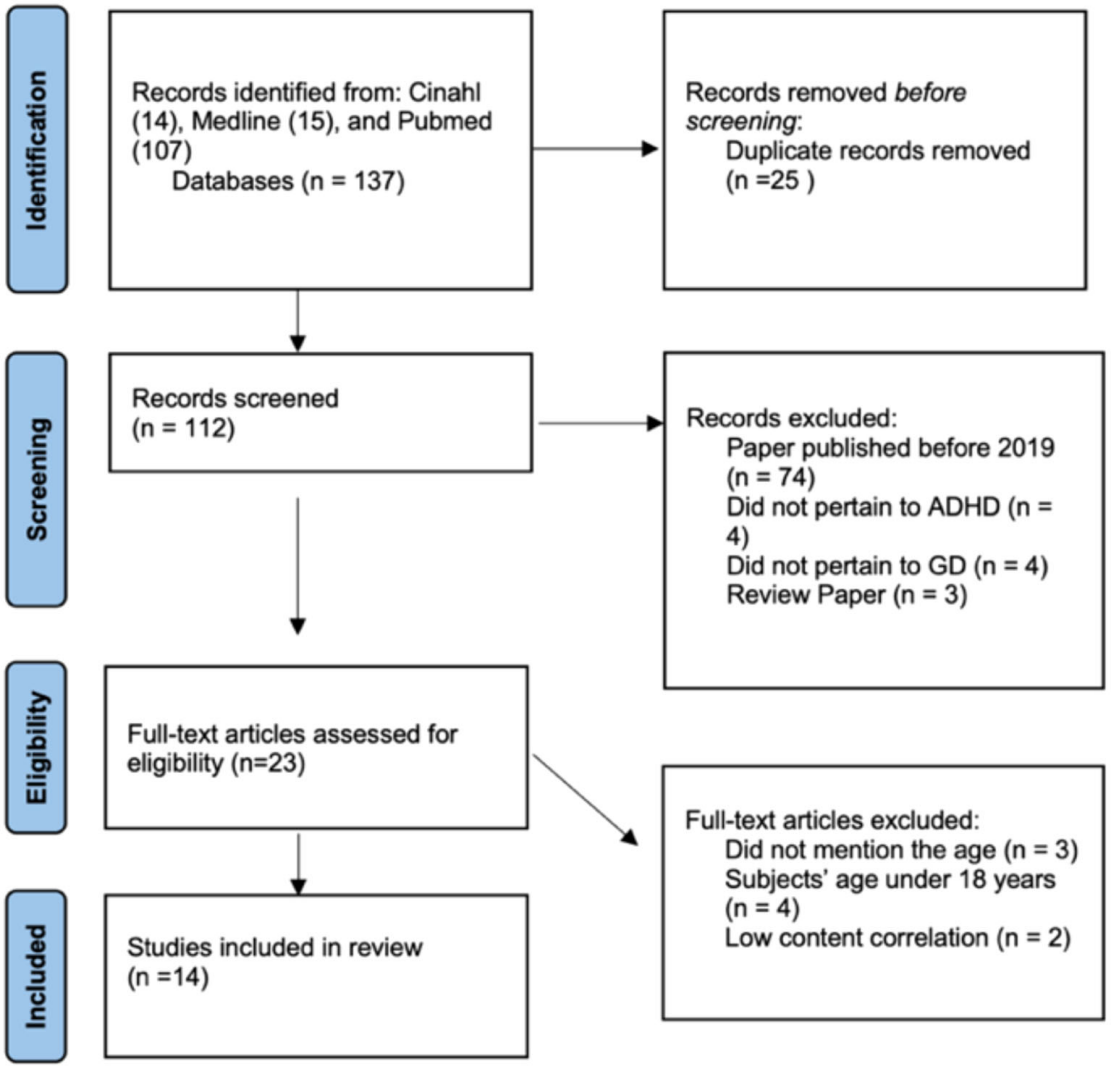
PRISMA diagram of literature search.

The initial search identified 14 articles from CINAHL, 15 articles from MEDLINE, and 107 articles from PubMed. After removing 25 duplicates, 112 articles were screened by abstract, of which 74 were published before 2019, 4 were unrelated to ADHD, and 4 were unrelated to IGD/GD, leaving 23 articles. Among these, 3 articles did not mention the sample age, 4 articles included samples with participants under 17 years old, and 2 were of low relevance to the theme of this review, resulting in 14 articles being included in the final full-text review (**Figure 1**; **Table 2**; 23, 24, 27, 28, 31–40). The following are some findings related to the three research questions.

**Table 2:** Summary of studies included in systematic literature review on the comorbidity between ADHD and IGD/GD.

No.	Year	Author	Country	Targeted group	N	Aim	Study design	Main findings	Exposure definition	Outcome definition
1	2019	Evren et al	Turkey	University students	N=1509	To evaluate the relationships between the severities of Internet Addiction (IA) and IGD symptoms with ADHD and aggression among university students.	Cross-sectional study. Online self-reported scales with Internet Addiction Scale (IAS) and Internet Gaming Disorder Scale - Short-Form (IGDS9-SF).	Probable ADHD is related to the severity of IA and IGD symptoms, as well as to aggression and depression.	ADHD symptoms/probable ADHD via ASRS;Aggression, depression as covariates.	IGD severity (IGDS9−SF total); Internet addiction severity (IAS total).
2	2019	Stavropoulos et al	Australia and the United States	Massively Multiplayer Online gamers aged 18 to 29.	N=621	To examine the moderating effects of gender in the association between ADHD and IGD across two nations.	Cross-sectional study. Online self-reported scales with ASRS and IGDS9-SF.	Compared with Australia, the males in the USA with hyperactive-impulsive and inattentive showing higher levels of disordered gaming.	ADHD symptom dimensions (inattention; hyperactivity−impulsivity) via ASRS.	IGD severity (IGDS9−SF total.)
3	2020	Stavropoulos et al	Australia and the United State	Massively Multiplayer Online gamers, with an average age of 24 years.	N=1032	To empirically assess the association between inattention and IGD, in the light of variable levels of vertical-individualism that reflects cultural inclinations towards independence, competitiveness, and hierarchy.	Cross-sectional study. Online self-reported scales with ASRS, IGDS9-SF, and Individualism-Collectivism Questionnaire.	Findings demonstrated an association between IGD and inattention, and additionally showed that this association was exacerbated by a more vertically-individualistic cultural orientation without significant gender differences.	Inattention (ASRS subscale); Vertical Individualism (Individualism–Collectivism).	IGD severity (IGDS9−SF total).
4	2020	Vally	the United Arab Emirates	Participants aged 18 to 33 years.	N=214	To examine the associations between inattention and impulsivity with IGD symptoms separately and to investigate whether gender moderates these relationships.	Cross-sectional study. Online self-reported scales with ASRS, IGDS9-SF, and reported their daily gaming duration along with several demographic characteristics.	Both inattention and impulsivity were significantly and positively associated with IGD symptoms. And gender did not moderate the relationships between inattention and impulsivity with IGD symptoms.	Inattention & impulsivity (ASRS subscales); daily gaming duration as covariate.	IGD severity (IGDS9−SF total).
5	2021	Chen et al	China	Freshmen from a local university	N=1236	To discover the mediating role of depressive symptoms and hopelessness on the relationship between ADHD and IGD symptoms.	Cross-sectional study. Self-reported scales with ASRS-5, IGDS9-SF, and Quick Inventory of Depressive Symptomatology-Self Report (QIDS-SR).	ADHD symptoms of college students impacted their IGD symptoms directly and indirectly via depressive symptoms and hopelessness.	ADHD symptoms (ASRS−5 total/subscales). Mediators: depression (QIDS−SR), hopelessness (BHS)	IGD symptoms (IGDS−9 dichotomous/count).
6	2021	Concerto et al	Italy	Video game players aged 18 to 55.	N=4260	To measure the prevalence of IGD in an adult population of video game players and to investigate the association between demographic variables, autism spectrum disorder (ASD) traits, ADHD severity, and IGD in adults.	Cross-sectional study. Online self-reported scales with ASRS, IGDS9-SF, and the Autism Spectrum Quotient (AQ).	Autistic traits and attention-deficit hyperactivity symptoms are positively associated with the severity of IGD in adult video game players	Autistic traits (AQ total); ADHD symptoms (ASRS total).	IGD severity (IGDS9−SF total; cutoff≥21).
7	2021	Evren et al	Turkey	E-sports players and university students	N=745	To explore the relationship between probable ADHD and playing Massively Multiplayer Online Role-Playing Games (MMORPGs), and how gaming motivations affect the severity of disordered gaming among young adults.	Cross-sectional study. Online self-reported scales with ASRS-v1.1, IGDS9-SF, and the Motives for Online Gaming Questionnaire (MOGQ). Data also collected from game development companies.	ADHD, the use of MMORPGs, and various gaming motivations together affect the severity of IGD.	Probable ADHD (ASRS−v1.1); MMORPG use; Gaming motivations (MOGQ).	Disordered gaming severity (IGDS9−SF total; cutoff).
8	2021	Ko et al	Taiwan	IGD (N=69), regular gamers (RGs, N=69), and non-gamers (N=69), aged 20 to 38 years, all with more than 12 years of education.	N=207	To evaluate the psychiatric comorbidities of patients with IGD. To assess the emotional intelligence (EI) and psychiatric symptoms of participants with IGD and psychiatric comorbidities, such Generalized Anxiety Disorder (GAD), Social Anxiety Disorder (SAD), and Major Depressive Disorder (MDD).	Cross-sectional study. Self-reported scales with scale for EI, depression, anxiety, and impulsivity. The diagnostic interview used meanwhile.	1. IGD was significantly associated with ADHD, GAD, and SAD, with ADHD being the most common comorbidity. 2. Participants with IGD had lower scores in all dimensions of EI, especially in self-control and well-being. 3. Participants with IGD and ADHD exhibited lower self-control, emotionality, and higher impulsivity. 4. Participants with IGD and emotional comorbidities (MDD, GAD, or SAD) had lower scores in all subscales of EI and higher scores for depression and anxiety.	ADHD diagnosis/symptoms; psychiatric comorbidities.	Group status (IGD/RG/NG); Emotional intelligence subscales; psychopathology scores.
9	2022	Masklavanou et al	Greek	Greek-speaking adults aged 18 and above.	N=515	To investigate the relationship between IGD and exercise as well as between IGD and ADHD. And examine whether symptoms of depression, anxiety, and stress mediate these relationships.	Cross-sectional study. Online self-reported scales through google with IGDS9-SF, Leisure-Time Exercise Questionnaire, Barkley Adult ADHD Rating Scale, and Depression, Anxiety and Stress Scale-21.	A negative correlation between IGD symptoms and leisure time exercise as well as a positive correlation between IGD symptoms and ADHD symptoms. Symptoms of depression were partially and significantly mediating the association between IGD symptoms and Attention deficit as well as the association between IGD symptoms and Impulsivity.	ADHD symptoms (Barkley Adult ADHD); Leisure−time exercise (LTEQ). Mediators: Depression/Anxiety/Stress (DASS−21.)	IGD severity (IGDS9−SF total).
10	2022	Kandeğer & Eğilmez	Turkey	Male university students	N=376	To examine the relationship between childhood trauma, dissociative experiences, and IGD in male university students with probable ADHD.	Cross-sectional study. Online self-reported scales with IGDS9-SF, ASRS, Wender Utah Rating Scale, Childhood Trauma Questionnaire, Dissociative Experiences Scale, Somatoform Dissociation Questionnaire, and a sociodemographic form.	Male university students with probable ADHD, determined by both childhood and current ADHD symptoms, had higher childhood trauma, dissociative experiences, and internet gaming disorder scores than those without.	Probable ADHD(ASRS + WURS);Childhood trauma (CTQ);Dissociative experiences (DES; SDQ).	IGD severity (IGDS9−SF total).
11	2023	Gul & Gul	Turkey	Medical students and resident doctors, aged 18 to 35.	N=274	To explore the relationships between ADHD, Sluggish Cognitive Tempo (SCT), demographic factors, and Internet Addiction (IA) and IGD among medical students and resident doctors.	Cross-sectional study. Self-reported scales with ASRS, Barkley SCT Scale, Young Internet Addiction Test-Short Form, the Digital Game Addiction Scale, and the sociodemographic form.	SCT symptoms increase the risk of Internet addiction and IGD even when ADHD symptoms are controlled.	ADHD symptoms (ASRS); Sluggish Cognitive Tempo—SCT (Barkley SCT:daydreaming, sluggishness).	IGD (Digital Game Addiction Scale) and Internet addiction (YIAT−SF).
12	2023	Hong et al	Korea	Clinical group seeking medical help for IGD (N=172), and general gamers (N=165), aged 17 to 29.	N=301	To compare the gaming patterns, accompanying psychopathology, and co-occurring psychiatric disorders, particularly ADHD, between clinical and general gamer samples. And explore the possibility of ADHD when diagnosing IGD/GD in patients with problematic Internet gaming.	Cross-sectional study. Data of demographics, gaming habits and patterns, accompanying psychopathology, and co-occurring psychiatric disorders were collected. Online data collecting for regular group, offline collecting for clinical group. The semi-structured diagnostic interview (Gaming Diagnostic Interview) used meanwhile.	The IGD (GD) group had a higher prevalence of ADHD compared to the general gamer group. There was a higher prevalence of IGD (GD) in the ADHD group compared to the non-ADHD group. The criterion of "functional impairment" had low diagnostic accuracy in participants with IGD or GD.	ADHD diagnosis/symptoms.	IGD/GD diagnosis; Criterion diagnostic accuracy; Psychiatric comorbidities.
13	2023	Lin et al	Taiwan	IGD (N=69), regular gamers (RGs, N=69), and non-gamers (N=69), aged 20 to 38 years, all with more than 12 years of education.	N+207	To evaluate the relationships between circadian typologies, insomnia, and IGD and how ADHD affects this relationship	Cross-sectional study. Self-reported scales with Diagnosis of adult ADHD (based on DSM-5 criteria), Composite Scale of Morningness (CSM), Pittsburgh Insomnia Rating Scale—20-item version (PIRS_20), Chen Internet Addiction Scale—gaming version (CIAS-G). The diagnostic interview used meanwhile.	ADHD exacerbated the eveningness preference and insomnia of individuals with IGD.	ADHD diagnosis (DSM−5); Circadian typology (CSM); Insomnia (PIRS−20).	IGD status/severity (CIAS−G); CSM/PIRS scores.
14	2024	Hawi & Samaha	Lebanon	University students aged 17 to 26.	N=348	To examine the mediating role of GD in the impact of ADHD on academic performance.	Cross-sectional study. Online self-reported scales with IGD-20, ASRS-v1.1, and GPA.	A positive correlation between ADHD symptoms and GD severity, which in turn correlates negatively with academic achievement.	ADHD symptoms (ASRS−v1.1 total).	Academic performance (GPA, self−reported).

### 3.2 RQ 1: is there a direct association between adult ADHD symptoms and the severity of IGD/GD?

All reviewed studies consistently indicate that ADHD symptoms, particularly inattention and hyperactivity-impulsivity, are associated with the incidence and severity of IGD/GD, suggesting that ADHD may be a significant risk factor for developing IGD/GD. In most studies, ADHD symptom severity (ASRS total/subscales) was treated as the exposure, while IGD severity (IGDS9−SF or IGD−20) or clinical IGD/GD diagnosis served as the outcome; two studies used semi−structured diagnostic interviews for IGD/GD (27, 35). One study found that the prevalence of ADHD among adult IGD/GD subjects (35.7%) was significantly higher than that among non-IGD/GD subjects (24.2%) (28). However, the causal pathways between adult ADHD and IGD/GD diagnoses have not been clearly established.

When ADHD and IGD/GD co-occur in an adult, they may jointly contribute to other symptoms. For instance, the co-occurrence of both conditions may further exacerbate IGD/GD symptoms through increased depressive symptoms and feelings of hopelessness (33), and other mental health issues such as obsessive-compulsive disorder and anxiety may also worsen (28, 36). Individuals with comorbid ADHD and IGD/GD exhibit lower self-control and higher impulsivity, as well as greater emotionality (24). Those with comorbidity have higher insomnia scores than individuals with IGD/GD alone (35). IGD/GD exacerbates the negative impact of ADHD symptoms on the academic performance of college students (28).

### 3.3 RQ 2: What factors potentially mediate the relationship between adult ADHD symptoms and IGD/GD symptoms?

**Figure 2:**
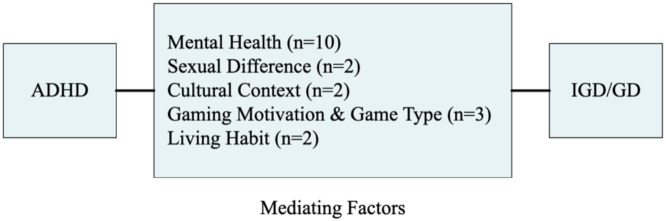
The mediating factors between ADHD symptoms and IGD/GD symptoms.

Five factors have been mentioned as potentially mediating the relationship between adult ADHD symptoms and IGD/GD symptoms: mental health, gender difference, cultural context, gaming motivation and game type, and living habit (**Figure 2**). It is worth noting that, given that the existing evidence mainly comes from cross-sectional studies, a causal mediation cannot be established, the pathways are better interpreted as associations or hypotheses.

Mental Health: Eight studies explicitly mention that depression and anxiety play important roles in the association between adult ADHD and IGD/GD (23, 24, 28, 33, 36–39). However, the causal relationship and specific mechanisms between them are rarely clarified. Is it that IGD/GD symptoms exacerbate ADHD symptoms through depression and anxiety, or the other way around? One study found that ADHD symptoms in first-year college students directly or indirectly affected their IGD/GD symptoms through depressive symptoms and feelings of hopelessness (33). ADHD patients might use gaming to cope with various negative emotions, thereby causing or worsening IGD/GD.

In addition to depression and anxiety, other mental health issues are also mentioned. Among adult male gamers, symptoms of autism spectrum disorder (ASD) are positively correlated with the severity of ADHD and IGD/GD (34). Male college students with ADHD symptoms scored higher in childhood trauma, dissociative experiences, and IGD/GD (37), suggesting that early life experiences might increase the risk of comorbid ADHD and IGD/GD. ADHD exacerbates insomnia symptoms in IGD/GD (35), and insomnia is often associated with depressive and anxious moods (41, 42). Social difficulties are also present (24, 35, 37).

Sexual Difference: As one of the basic demographic indicators, the gender ratio of subjects was mentioned in almost all studies. Two studies did not provide specific data but indicated that this information was collected (27, 39), and one study included only male subjects (37). However, most of the time, gender distribution was only considered insignificant background information. Only two studies examined in detail the role of gender as a variable in the association between adult ADHD and IGD/GD, and both had relatively balanced numbers of male and female subjects. Among Massively Multiplayer Online (MMO) players in the United States, male ADHD subjects exhibited more IGD/GD symptoms than female ADHD subjects (31), with 457 subjects, 57.98% male and 42.02% female. Another study using the PROCESS macro for SPSS found that gender did not significantly moderate the association between ADHD and IGD/GD, with 214 subjects, 44.9% male and 55.1% female (40). Although this study was conducted in the UAE, 28% of the subjects were from overseas, so the geographical qualifier “the United Arab Emirate” cannot be added to the conclusion.

An interesting phenomenon worth noting is that in 11 studies that presented the gender ratio and number of subjects, 7 studies had more male subjects than female subjects (24, 31, 34–36, 38), accounting for 63.6% in the studies which provide gender ratio. This may suggest some cultural biases, with more males playing games and females spending more time on social media than gaming (43, 44). Additionally, males may have a stronger personal connection to gaming topics and thus be more willing to participate in such studies (28). Of course, the gender ratio of subjects is also related to recruitment channels and occupational categories. For example, in two studies targeting college students, medical students, and residents, the number of female subjects overwhelmingly exceeded that of males (23, 33).

Cultural Context: Scholar Stavropoulos and colleagues (31, 32) introduced the dimension of vertically-individualistic culture in their two studies to explore the association between adult ADHD and IGD/GD. “Vertical individualism,” as a cultural characteristic, emphasizes determining social values through personal achievements and competition. Such a cultural tendency may weaken social connectedness and increase feelings of isolation, prompting individuals to adopt pathological behaviors (such as addictive behaviors) to regulate emotions (45). This cultural tendency is considered to be higher in the United States than in Australia (46, 47). Therefore, it can be said that individuals or American male ADHD players with a more “vertical individualism” inclination are more likely to fall into the risk of IGD/GD (31, 32).

Cultural differences have been considered in studies of other addictive behaviors, such as food addiction (48), social addiction (49, 50), and alcohol addiction (51, 52). Although there are currently not many studies introducing cultural context as a variable in the association between adult ADHD and IGD/GD, research in this direction can help develop more culturally sensitive treatments and interventions in cross-cultural settings. Particularly in some cultural contexts, mental health issues may be stigmatized (53, 54), leading to feelings of shame among patients (55–57), which could further exacerbate addictive behaviors.

Gaming Motivation & Game Type: Among the 14 articles we reviewed, three addressed gaming motivation and game type. A study on massively multiplayer online role-playing games (MMORPGs) players found that individuals with potential adult ADHD preferred playing multiplayer online battle arena games, social network games, music games, MMORPGs, sports/car games, and horror-themed/survival games. Additionally, four gaming motivations were particularly significant among those with potential adult ADHD: coping/escape, recreation, fantasy, social and competition (using 58 classification of gaming motivations). The manifestation of potential ADHD, usage of MMORPGs, and the four gaming motivations collectively predicted the severity of IGD/GD symptoms (39). However, the subjects of this study were university students in Turkey, and different regions (59, 60) and occupations (61, 62) may have varying influences on the formation of gaming motivations. The intersectional relationships among these factors should be considered more cautiously.

Two studies focused on Massively Multiplayer Online (MMO) players, suggesting that due to the structural characteristics and immersive nature of MMO games, players with more severe ADHD symptoms are more likely to exhibit IGD/GD behaviors in MMO games (31, 32). However, these two studies only analyzed the unidirectional impact of ADHD on IGD/GD within MMOs and did not evaluate the reverse effect of IGD/GD on ADHD.

Living Habit: Circadian typologies and leisure exercise were mentioned in two of the reviewed articles, respectively. One study found that IGD/GD exhibited a stronger eveningness preference, with the severity of IGD/GD being positively correlated with insomnia. Moreover, ADHD exacerbated the severity of circadian rhythm disruptions and insomnia in IGD/gd patients (35). Many previous studies have also discussed the relationship between circadian rhythm disruptions and internet addiction, most notably the impact of blue light emitted by phone and computer screens, which can inhibit melatonin secretion, delay the biological clock, and disrupt circadian time structure (63).

The other study did not directly discuss leisure exercise as a mediating factor between ADHD and IGD/GD. Instead, it analyzed the correlations between exercise, IGD/GD, mental health, and ADHD. There was a negative correlation between exercise and IGD/GD symptoms; individuals with more IGD symptoms tended to engage less in leisure-time exercise. There was also a negative correlation between exercise and symptoms of depression, anxiety, and stress; individuals who exercised more had fewer of these symptoms. A positive correlation was found between ADHD symptoms and IGD/GD symptoms. Exercise had a significant impact on alleviating symptoms of both ADHD and IGD/GD (36).

### 3.4 RQ 3. How is academic performance in adult ADHD affected by IGD/GD?

Of the 14 articles we reviewed, six included college students (23, 28, 33, 37–39). But five of them simply mentioned that ADHD and IGD/GD could have a negative impact on academic performance. Only one paper (28) discussed in detail the relationship between ADHD, IGD/GD and GPA through questionnaire and data analysis, and found that IGD played a mediating role in the negative impact of ADHD symptoms on academic performance. The positive correlation between ADHD symptoms and IGD severity and the negative correlation between IGD severity and academic achievement were further verified.

## 4 Discussions

### 4.1 The geographical region from which the study came and the year of publication

The 14 articles come from 9 geographical regions. Our statistics consider the regions where the research was conducted rather than the first author’s location or origin. An obvious fact is that 10 studies come from Asia, constituting an overwhelming 71%, with contributions from 9 different first authors. Notably, Turkey, which we categorize as part of Asia due to 97% of its area being in Asia, has the most related research, with 4 articles (29% of the total), two of which are from the same first author. Europe has one study each from Italy and Greece, accounting for 14%. Australia and the United States are discussed together, contributing 2 articles in total, both published by the same first author, with research subjects indicated to be from both locations. Geopolitically, Asia has a particularly rich scholarly presence in this field. It is important to emphasize that this represents the situation since 2019. From 2019 to 2024, the number of related publications has shown a fluctuating trend: 2 articles each in 2019 and 2020, peaking at 4 articles in 2021, dropping to 2 in 2022, rising again to 3 in 2023, and 1 article published in 2024. This indicates that there is a certain volatility in the research activity in this field.

### 4.2 Generation of bias from study design

**Figure 3:**
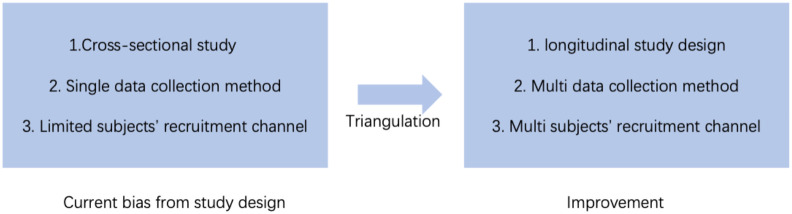
The current bias from study design and potential improvement.

All of the studies reviewed were cross-sectional studies, which limits the possibility of determining causality for variables. Cross-sectional studies do have various advantages, such as relatively fast and low cost, easy hypothesis generation, etc. (64), they can collect large amounts of data in a relatively short period of time, and are very useful for initial exploration of relationships between variables (**Figure 3**). However, there are significant limitations to this type of research approach, particularly in establishing causation. Because cross-sectional studies only collect data at a single point in time, they cannot reveal chronological relationships between variables, which makes it difficult to determine whether ADHD symptoms cause IGD or if IGD symptoms in turn exacerbate ADHD.

To address this limitation, future studies should consider a longitudinal study design. This design allows researchers to follow the same group of subjects over a longer period of time, allowing for a clearer view of how variables change and interact over time. Longitudinal studies can not only help determine the chronological relationship between adult ADHD and IGD, but also explore the underlying causal mechanisms. For example, by tracking data at different points in time, researchers can more accurately analyze how IGD affects the academic performance of adults with ADHD, revealing whether IGD is somehow contributing to the decline in their academic performance. In addition, longitudinal studies can help identify mediating and moderating variables that influence the relationship between ADHD and IGD at different points in time, providing a more complete understanding.

In addition, data from 11 studies were collected from self-reported scales, commonly used scales include Adult ADHD Self-Report Scale (ASRS) and Internet Gaming Disorder Scale-Short Form (IGDS9-SF). Although these scales are widely used in clinical and research Settings and provide a degree of convenience and standardization, their results can be affected by subject subjectivity bias. The self-report scale relies on an individual’s self-perception and recall of their own symptoms and behaviors, which can result in the accuracy of the report being affected by the individual’s cognitive biases, memory errors, or the effects of social expectations. For example, some subjects may underestimate or overestimate the severity of their symptoms to conform to their perceptions of societal expectations or to avoid negative evaluations.

Of these studies, only three used diagnostic interviews in conjunction with the scale (24, 27, 35). Diagnostic interviews, through face-to-face communication with a subject by a professional clinician, can provide more detailed and accurate diagnostic information and help mitigate possible biases in self-reports. Therefore, the method of combining scale and diagnostic interview can improve the accuracy and reliability of data.

Future studies may consider using a combination of multiple data collection methods to improve the comprehensiveness and accuracy of the data. For example, in addition to self-report scales and diagnostic interviews, researchers may use behavioral observations, third-party reports (such as family or friend evaluations), and physiological measures (such as electroencephalography or imaging techniques). These diverse data collection methods not only provide a more comprehensive assessment of ADHD and IGD symptoms, but also help verify consistency between different data sources, thereby increasing the credibility of the findings. By combining multiple data collection methods, researchers were able to capture the complex relationship more accurately between ADHD and IGD, reducing the biases and limitations of a single approach. This will contribute to a deeper understanding of the characteristics and interactions of these disorders, and provide a more solid scientific basis for clinical diagnosis and treatment.

In addition, the recruitment channels of subjects may be directed to specific populations, with several articles recruiting subjects via the Internet or social media (27, 31, 34, 36, 40). Although this method of recruitment is convenient and can quickly attract a large number of subjects, it also has obvious limitations. First, subjects recruited via the Internet or social media tend to be those who use these platforms heavily. This means that people who do not frequently surf the web or are not interested in social media may be excluded, resulting in a limited representation of the sample.

This method of recruitment may lead to sample bias. For example, people who use the Internet and social media heavily may have certain characteristics that differ from people who use these platforms less frequently. They may have higher Internet dependence, different patterns of social behavior, and specific mental health conditions. These characteristics may have a potential impact on the study results, making it difficult to generalize the results to the wider population. In addition, subjects recruited online may be more inclined to share their online behavior and mental health issues, which can lead to bias in the data.

In order to improve the generality and representativeness of the findings, future studies should consider using multiple recruitment channels. In addition to the Internet and social media, researchers can recruit through traditional offline methods, such as posting recruitment advertisements in schools, medical institutions, community centers, etc., or mailing invitations. In addition, researchers can work with various community organizations to directly contact individuals with different backgrounds and lifestyles to ensure sample diversity and representation.

### 4.3 Potential future research directions

**Figure 4:**
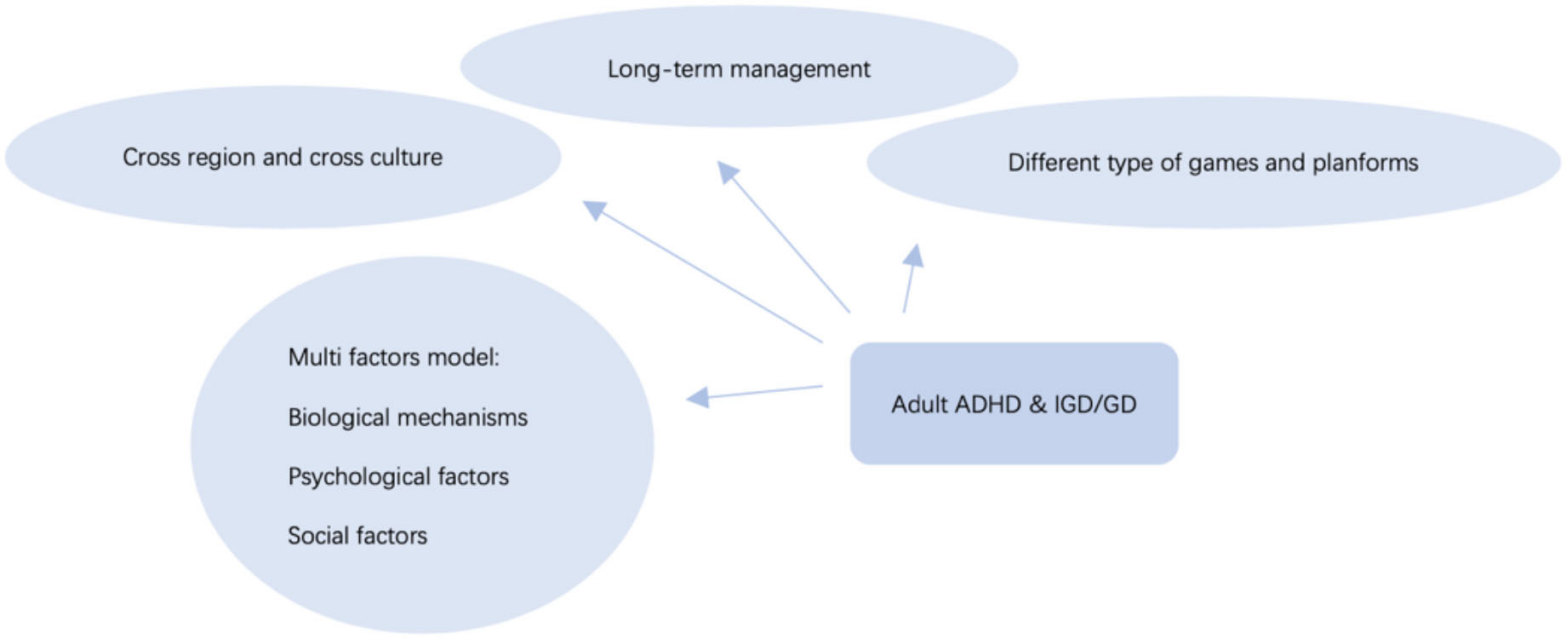
The potential future research directions.

Future studies of adult ADHD and IGD comorbidity should consider establishing a multi-factor model to explore the interaction and relative effects of biological, psychological and social factors on ADHD and IGD. These factors include, but are not limited to (**Figure 4**):

- Biological mechanisms: Future research could provide insight into the biological basis of the link between ADHD and IGD through neuroimaging studies, genetic studies, and analysis of structural and functional changes in the brain. Specifically, researchers can use functional magnetic resonance imaging (fMRI), positron emission tomography (PET) and other techniques to look at activity levels and connectivity patterns in different brain regions in ADHD and IGD patients. In addition, genomic studies can reveal the genetic predisposition associated with both disorders, thereby providing the basis for personalized treatment.
- Psychological factors: Psychological factors play an important role in the comorbidity of ADHD and IGD. Future studies can explore how individual psychological characteristics such as emotional regulation, impulse control, and cognitive function affect the interaction of these two disorders. Through longitudinal study design, researchers can observe changes in psychological factors at different stages of development and their role in the development of comorbidity.
- Social factors: Social factors cannot be ignored either. Studies can examine the effects of family environment, social support, and school and work environments on ADHD and IGD. For example, different parenting styles, the strength of social support networks, and stress levels in the work environment may all influence the severity and progression of both disorders to some extent. In addition, education of related diseases is also an area of concern, by increasing public awareness of ADHD and IGD, early identification and intervention can be made to reduce symptoms.

In view of the above factors, this study raises some potential research questions in the future:

1. What are the similarities and differences in brain structure and function between ADHD and IGD patients? Can neuroimaging studies reveal the role of specific brain regions or neural networks in these two disorders?
2. What genetic factors are associated with the comorbidity of ADHD and IGD? Can genomics studies identify specific genetic variants or genetic markers?
3. What is the role of emotion regulation and impulse control in ADHD and IGD comorbidity? Can these psychological characteristics be used as targets for intervention?
4. How does cognitive impairment affect the relationship between ADHD and IGD? Can cognitive behavioral therapy effectively improve the symptoms of comorbidities?
5. What role do family environment and social support play in the comorbidity of ADHD and IGD? What are the effects of different family education methods on comorbidity?

In addition, most current studies focus on specific countries or regions, while large-scale multi-regional cross-cultural studies can verify the research results under different cultural backgrounds and improve the universality and external validity of the research. Different cultural backgrounds may have significant differences in individuals’ play behaviors, family and social support systems, and perceptions and attitudes toward ADHD and IGD, all of which may influence the findings. Therefore, cross-cultural research can not only enrich our understanding of ADHD and IGD, but also provide intervention and treatment strategies tailored to different cultural contexts.

Research can also explore different types of games (e.g. Multiplayer Online Battle Arena, First-Person Shooter, Role-Playing Game) and different platforms (e.g. mobile devices, game consoles) Effects of consoles and personal computers on IGD and ADHD symptoms. Different types of games and platforms may have different effects on player psychology and behavior, thus playing different roles in the comorbidity mechanisms of ADHD and IGD. By comparing the effects of different game types and platforms, researchers can identify high-risk and low-risk gaming behaviors and develop prevention and intervention measures accordingly.

Finally, although existing studies have pointed to comorbidities and some mediating factors in adults with ADHD and IGD, there have been few studies on effective prevention and treatment, and there has been no long-term follow-up to investigate recurrence rates and influencing factors. Integrated treatment options and long-term management strategies for comorbidities of these two disorders are valuable. Future studies could design and test multiple interventions, such as medication, cognitive behavioral therapy, family therapy, etc., and evaluate their effects with long-term follow-up. This not only helps to reduce symptoms, but also improves patients’ quality of life and social functioning.

## 5 Conclusion

This systematic review on interactive Impact of Internet Gaming Disorder and Attention Deficit Hyperactivity Disorder to adult learning group is based on a sample of 14 papers out of 137reviewed. The review found a direct association between adult ADHD symptoms and the severity of IGD/GD. It identified five potential mediating factors in the relationship between adult ADHD symptoms and IGD/GD symptoms: mental health, gender difference, cultural context, gaming motivation and game type, and living habit; IGD/GD has a negative effect on the academic performance of adults with ADHD. This paper also has limitations: the databases searched were limited, the selected articles were few, and only studies from the past five years were reviewed.

## Data Availability

The original contributions presented in the study are included in the article/**Supplementary Material**. Further inquiries can be directed to the corresponding author/s.

## Supplementary material

Supplementary Table 1 Used the Newcastle–Ottawa Scale (NOS) to assess the quality of the 14 studies.
