# Supplementary material for: Internet gaming disorder, attention deficit hyperactivity disorder and learning in adults: a systematic review
Source: Front Psychiatry. 2026 Jan 22;16:1735922. doi: 10.3389/fpsyt.2025.1735922 (PMC12872851; doi:10.3389/fpsyt.2025.1735922)
Supplement: Supplementary Table 1 — Used the Newcastle–Ottawa Scale (NOS) to assess the quality of the 14 studies. [file DataSheet1.pdf]

Study	Selection					Comparability		Outcome			Total (10/10)	Note
	Representativeness	Sample Size Justification	Non Respondents	Exposure Ascertainment	Exposure Quality	Comparability Core	Comparability Additional	Outcome Assessment	Outcome Definition	Statistics Appropriate		
Evren et al (2019)	0	0	0	1	0	1	1	1	1	1	6/10	ASRS; ANCOVA adjusted; IGDS9-SF cutoff; proper stats.
Stavropoulos et al (2019)	0	0	0	1	0	1	1	1	1	1	6/10	ASRS; multivariable/interaction; IGDS9-SF cutoff; proper stats.
Stavropoulos et al (2020)	0	0	0	1	0	1	1	1	1	1	6/10	ASRS; VI moderation (sex/age); IGDS9-SF; proper stats.
Vally (2021)	0	0	0	1	0	1	1	1	1	1	6/10	ASRS; hierarchical/PROCESS (sex/age/time); IGDS9-SF cutoff.
Chen et al (2021)	0	0	0	1	0	1	1	1	1	1	6/10	ASRS-5; PROCESS mediation (sex/age); IGDS-9 DSM threshold.
Concerto et al (2021)	0	0	0	1	0	1	1	1	1	1	6/10	ASRS; regression (demo + AQ); IGDS9-SF cutoff 21.
Evren et al (2021)	0	0	0	1	0	1	1	1	1	1	6/10	ASRS; ANCOVA/regression; motives/MMORPG; IGDS9-SF.
Ko et al (2021)	0	0	0	1	1	1	1	1	1	1	7/10	Clinical interviews ADHD/IGD; diagnostic accuracy; proper stats.
Masklavanou et al (2022)	0	0	0	1	0	1	1	1	1	1	6/10	BAARS/ASRS; hierarchical + Sobel; IGDS9-SF.
Kandeğer & Eğilmez (2022)	0	0	0	1	0	1	1	1	1	1	6/10	ASRS+WURS; hierarchical (CTQ); IGDS9-SF.
Gul & Gul (2023)	0	0	0	1	0	1	1	1	1	1	6/10	ASRS+SCT; SEM paths; DGAS-7/YIAT-SF.
Hong et al (2023)	0	0	0	1	1	1	0	1	1	1	6/10	Clinical interviews; ADHD & IGD/GD; accuracy analysis.
Lin et al (2023)	0	0	0	1	1	1	0	1	1	1	6/10	Clinical ADHD/IGD; regression (sex/age); CSM/PIRS_20.
Hawi & Samaha (2024)	0	0	0	1	0	1	0	1	1	1	5/10	ASRS v1.1; regression/SEM; IGD-20 threshold 71.

Scoring Criteria:
Exposure Quality = 1: Requires clinical interview/diagnosis or multi-source verification; self-report scale only = 0.
Comparability Core = 1: Controls ≥1 core confounder (e.g., sex/age) in the main model or uses stratified analysis.
Comparability Additional = 1: Controls ≥1 additional key confounder in the same primary model (e.g., depression/anxiety, gaming time, sleep, SES), or path/structural models that explicitly include multiple relevant confounders.
Outcome Definition = 1: Uses a clear threshold or DSM/ICD diagnostic criterion and reports classification/prevalence (e.g., IGDS9-SF ≥36, IGD-20 ≥71, or clinical diagnosis).
Statistics Appropriate = 1: Methods fit aims and are sufficiently reported (e.g., regression/ANCOVA/mediation/moderation; SEM with fit indices); no major methodological errors.
